# Adipose Tissue-Derived Extracellular Vesicles: A Promising Biomarker and Therapeutic Strategy for Metabolic Disorders

**DOI:** 10.1155/2023/9517826

**Published:** 2023-12-26

**Authors:** Wenhui Liu, Tianyan Liu, Qingyu Zhao, Junqiu Ma, Jiajia Jiang, Hui Shi

**Affiliations:** ^1^Aoyang Institute of Cancer, Affiliated Aoyang Hospital of Jiangsu University, 279 Jingang Road, Zhangjiagang, Suzhou 215600, Jiangsu, China; ^2^Zhenjiang Key Laboratory of High Technology Research on sEVs Foundation and Transformation Application, School of Medicine, Jiangsu University, 301 Xuefu Road, Zhenjiang 212013, Jiangsu, China; ^3^Center of Laboratory Medicine, Affiliated Aoyang Hospital of Jiangsu University, 279 Jingang Road, Zhangjiagang, Suzhou 215600, Jiangsu, China; ^4^Department of Nephrology, Affiliated Aoyang Hospital of Jiangsu University, 279 Jingang Road, Zhangjiagang, Suzhou 215600, Jiangsu, China

## Abstract

Adipose tissue plays an important role in systemic energy metabolism, and its dysfunction can lead to severe metabolic disorders. Various cells in adipose tissue communicate with each other to maintain metabolic homeostasis. Extracellular vesicles (EVs) are recognized as novel medium for remote intercellular communication by transferring various bioactive molecules from parental cells to distant target cells. Increasing evidence suggests that the endocrine functions of adipose tissue and even the metabolic homeostasis are largely affected by different cell-derived EVs, such as insulin signaling, lipolysis, and metabolically triggered inflammation regulations. Here, we provide an overview focused on the role of EVs released by different cell types of adipose tissue in metabolic diseases and their possible molecular mechanisms and highlight the potential applications of EVs as biomarkers and therapeutic targets. Moreover, the current EVs-based therapeutic strategies have also been discussed. This trial is registered with NCT05475418.

## 1. Introduction

Adipose tissue is composed of several cell types, including adipocytes, immune cells, endothelial cells, and stem cells [1]. It is an important organ of energy metabolism in the human body. Adipose tissue is able to regulate systemic metabolism through the uptake of glucose and fatty acids as well as release a variety of biologically active molecules, such as adipokines, hormones, and extracellular vesicles (EVs) [2–4]. Dysfunctional adipose tissue promotes dysregulation of metabolism, which can lead to obesity, cardiovascular disease, and diabetes [5–9]. At the same time, in the presence of dysregulated metabolism, the overproduction of proinflammatory adipokines and the decreased expression of anti-inflammatory adipokines further contribute to the increase in adipose tissue volume, as well as adipocyte damage and degeneration, which exacerbate obesity and insulin resistance, among others [10–12]. In addition to adipokine dysregulation, EVs serve as a natural intercellular and interorgan communication tool. They are capable of carrying a variety of bioactive substances for information exchange. Early evidence suggests that adipose tissue-derived EVs have been found to play an important role in disorders of metabolism [13–17]. They can function as insoluble mediators in organ communication and regulate receptor organ function. EVs have been reported to act as mediators of communication between adipose tissue and other peripheral tissues, such as liver and skeletal muscle, and are involved in the development of non-alcoholic fatty liver disease (NAFLD) and diabetes mellitus type 2 (T2DM) [18]. In this review, we focused on the role of EVs of different cellular origins in adipose tissue in metabolic diseases (see Table 1), pointing out that EVs of different cellular origins in adipose tissue are expected to be promising biomarkers and therapeutics for the diagnosis and treatment of metabolic diseases, in order to facilitate the development of new therapeutic strategies.

## 2. The Role of Adipose Tissue in Metabolic Diseases

### 2.1. Adipose Tissue and Obesity

Obesity is a strong risk factor for metabolic diseases, which significantly reduces the quality of life and imposes a huge burden on society [47–49]. Obesity-associated hypoxia is thought to be a key initiator of adipose tissue dysregulation and inflammation, which can activate hypoxia-inducible factor (HIF-1*α*). HIF target genes are widely involved in cellular functions including glucose utilization, angiogenesis, apoptosis, and extracellular matrix remodeling and inflammation [50–52]. Upregulation of inflammatory factors in adipose tissue further exacerbates obesity symptoms. Numerous proinflammatory mediators are present in adipocytes and macrophages, such as interleukin-6 (IL-6), tumor necrosis factor-a (TNF-a), C–C motif chemokine ligand 2 (CCL2), inducible nitric oxide synthase (iNOS), and others. These various cytokines in turn amplify and sustain inflammation by further recruiting, activating, and inducing macrophage proliferation, which drives dysregulation of glucose, lipid, and energy metabolism [48]. However, unresolved inflammation is usually associated with the progression of fibrosis in pathological states. Some current evidence suggests a role for macrophages in obesity-induced fibrosis of white adipose tissue. Toll-like receptor 4 (TLR4) activation in macrophages recruits macrophage-induced c-type lectins, which promote extracellular matrix production as well as fibroblast proliferation and differentiation pathways [53]. The three aspects of obesity-associated hypoxia, inflammation regulation, and fibrosis progression are not isolated but are interconnected and mutually reinforcing.

### 2.2. Adipose Tissue and Abnormal Lipid Metabolism-Related Cardiovascular Diseases

Current epidemiologic studies have revealed that obese individuals, especially those with insulin resistance, share a higher risk in cardiovascular functional disorder. Obesity- and insulin resistance-related cardiovascular disease has been observed in all age groups, including children [54–56]. There is a close association between perivascular adipocytes and macrophages that modulates adipose tissue-associated inflammation. Vascular injury is able to induce the accumulation of perivascular adipose tissue with macrophages; meanwhile, neuromodulatory protein 4 (Nrg4) released by beige adipocytes promotes the activation of inflammation-suppressing macrophages, which in turn attenuates the inflammatory response to inhibits the progression of atherosclerosis [57]. Under pathological conditions, the abnormal metabolism of adipocytokines released by visceral adipose tissue hinders the normal function of various organs and affects cardiovascular health. For example, lipocalin and reticulin, which are released by visceral adipocytes, have been shown to prevent atherosclerosis by increasing nitric oxide production by endothelial cells, inhibiting inflammation of endothelial cell origin, and reducing foam cell formation [58]. A recent study similarly revealed the potential endocrine and paracrine effects of adipose tissue on the cardiovascular system. Akawi et al. [59] reported that sphingolipid released in visceral adipose tissue was higher in obese patients than in lean persons. It was further determined that visceral adipose tissue releases sphingolipids via ceramide 16:0 (Cer16:0)-rich EVs, and a series of correlation analyses were performed to demonstrate the positive correlation between circulating Cer16:0 and hypersensitivity C-reactive protein as well as reduced vasodilation in humans [59].

### 2.3. Adipose Tissue and NAFLD

NAFLD is defined as the accumulation of fat in liver in patients who do not consume excessive alcohol, which is the hepatic manifestation of metabolic diseases [60]. Studies have shown that increased expression of inflammatory genes and macrophage activation in visceral and subcutaneous adipose tissue of NAFLD patients correlate with progression of simple steatosis of fibrosis [61]. Furthermore, Falkevall et al. [62] utilized a vascular endothelial growth factor-B (VEGF-B) antagonist to inhibit the progression of white adipose tissue inflammation, revolved insulin resistance in white adipose tissue, and reduced hormone-sensitive adiponectin activity, resulting in improvement of NAFLD. To sum up, adipose tissue plays an important role in the liver in recent years, which is expected to provide a new target for the diagnosis and treatment of NAFLD caused by abnormal metabolism of adipose tissue.

### 2.4. Adipose Tissue and T2DM

The increased prevalence of obesity may be the main reason for T2DM [63]. Numerous studies are targeting adipose tissue to treat T2DM. Researchers observed mitochondrial dysfunction in white adipose tissue cells can increase oxidative and nitrosative stress in white adipose tissue. Dynamin-related protein 1 (Drp-1) is a key mediator of mitochondrial fission, successful regulation of the mitochondrial network by blocking Drp-1 ameliorated white adipose tissue abnormalities in obese and diabetic patients [64]. In addition, B-1a cells, a subpopulation of B lymphocytes, have been shown to be the major producers of Interleukin-10 (IL-10) in visceral adipose, contributing nearly half of the IL-10 *in vivo*. B cell-deficient mice resulted in a rapid improvement in insulin resistance and glucose tolerance [65]. Besides B cells, modulation of angiogenic signal in endothelial cells in adipose tissue is also considered a promising strategy to ameliorate obesity and insulin resistance. For example, activation of VEGF-A leads to increased adipose vascular system and reduced hypoxia [66, 67]. Recent studies have also addressed that adipose tissue-derived EVs from obese and unhealthy metabolically patients can reduce insulin resistance in myotubes and hepatocytes [68], which in turn promotes the progression of T2DM. Meanwhile, a lately study found that proteins and lipids are exchanged between endothelial cells and adipocytes of adipose tissue via EVs, these EVs are regulated by nutritional status [46]. For the past few years, a large amount of studies highlight the critical role of adipose tissue-derived EVs, especially EVs in the regulation of organismal metabolism and emphasize the importance of elucidating their underlying molecular and cellular mechanisms. In the following, we will elucidate in detail the roles and mechanisms of EVs from different cellular sources of adipose tissue in metabolic diseases.

## 3. Role of Adipose Tissue-Derived EVs in Metabolic Diseases

### 3.1. Extracellular Vesicles

**Figure 1:**
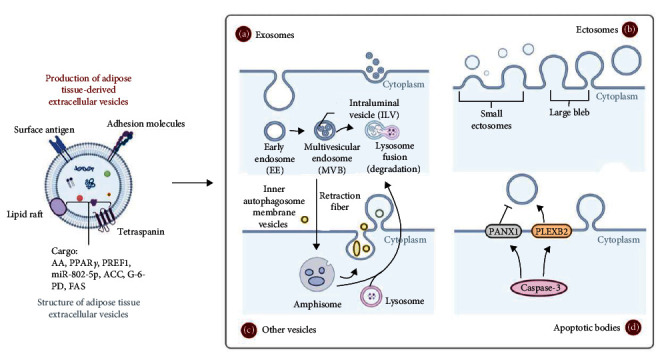
Structure and production of adipose tissue-derived extracellular vesicles. This figure was created by using BioRender (https://biorender.com/).

EVs are nanoparticles of lipid bilayers 50–1,000 nm in size [69], which are proved to be important way for cell–cell crosstalk and message exchanging [70–75]. EVs can be released almost by all types of cells and are found in a variety of body fluids including blood, saliva, urine, breast milk, and amniotic fluid [76, 77], thereby affecting distant tissues or remaining near the site of release to promote autocrine and or paracrine signal. Based on their biogenesis pathways and size distribution, recent studies have further categorized EVs into exosomes, ectosomes, apoptotic vesicles, and other types of vesicles (e.g., migratory bodies) [78–81]. The formation of small EVs, particularly the exosomes, is widely recognized as three successive steps: the plasma membrane of the cell invaginates for the first time to form endocytic vesicles, multiple endosomes fuse to form early endosomes, and intraluminal multivesicular bodies (MVBs) are formed in the early endosomes that enclose the intracellular materials. Lastly, MVBs fuse with the plasma membrane, releasing intraluminal vesicles into the extracellular space [81, 82]. Ectosomes originate as blisters or bumps severed from the plasma membrane, and nowadays immunoelectron microscopy has made it possible to visualize small ectosomes, which are removed from the plasma membrane through a signaling pathway involving the inhibitory structural domain-containing protein 1 (ARRDC1) [81, 82]. Signaling pathway germinating from the plasma membrane, and these ARRDC1-mediated microvesicles can be expressed on the cell surface of ARRDC1-mCherry94 and thus can be detected by immunoelectron microscopy containing anti-mCherry94 [83]. Apoptotic vesicles arise from the orderly fragmentation of apoptotic cells, and caspase 3 substrates play a key role in the formation of apoptotic vesicles, including Rho-associated protein kinase (ROCK1) and other regulatory factors such as pannexin 1 (PANX1) and plexin B2 (PLEXB2) [84]. In recent years, researchers have discovered that along the contractile fibers of migrating cells, there are projections containing internal vesicles, these structures are called “migrants” and can be released outside the cell as a large EVs [85] (Figure 1).

### 3.2. EVs Derived from Adipose Tissue

**Figure 2:**
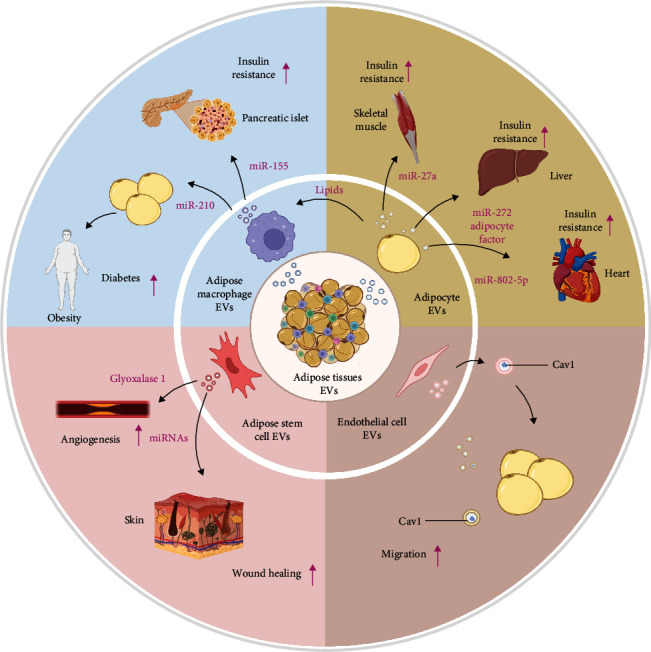
Classification of adipose tissue-derived EVs and their mediation of interorgan crosstalk in metabolic diseases. The composition of adipose tissue EVs can be altered in different physiological states to affect organs in pathological conditions such as obesity or diabetes, and several studies have focused on the role of adipose tissue EVs in obesity-associated insulin resistance. This figure was created by using BioRender (https://biorender.com/).

Functional EVs can be obtained from adipose tissue. Zhou et al. [19] isolated EVs from brown adipose tissue to treat the obese mice. They found EVs gained from brown adipose tissue significantly mitigated the syndrome in high-fat-diet mice [19]. In response to such phenomenon, the researchers further revealed Nucleophosmin 3 (NPM3) transferred by EVs derived from brown adipose tissue regulate the stability of PR domain-containing 16 (PRDM16) mRNA and enhance the expression of browning-related genes, indicating EVs from adipose tissue might represent a promising therapeutic strategy for metabolic diseases [20]. In addition to NPM3, the importance of miRNA in EVs derived from adipose tissue has been established through adipose-specific knockout of dicer, the critical component of miRNA biogenesis [21, 86]. However, the contents of adipose tissue derived EVs are not invariable. With the progression of obesity, the hypoxic conditions increased the proteins associated with lipid synthesis, such as acetyl coenzyme a carboxylase, glucose 6 phosphate dehydrogenase, and fatty acid synthase in adipose tissue EVs at three to four times the level of other types of EVs [87]. At the same time, obesity status affects not only the contents of adipose tissue EVs but also the biogenesis of EVs [88, 89]. As the state of obesity affects the contents and biosynthesis of adipose tissue EVs, these changes can further result in metabolic disorders in the body. Clinical studies have shown that subcutaneous adipose tissue EVs from obese patients are enriched in fatty acid oxidation-related proteins, whereas in animal model studies, injection of adipose-derived EVs in ob/ob mice induced insulin resistance, promoted the differentiation of bone marrow-derived monocytes into macrophages, and increased release of proinflammatory factors, exacerbating the progression of associated metabolic diseases [90, 91]. Overall, published literature underscored that adipose tissue EVs can be beneficial or detrimental depending on whether the donor is healthy or has a metabolic disease. These results identify EVs as potential future diagnostic biomarkers and therapeutic strategies for metabolic diseases. Besides tissue-derived EVs, for the past few years, an increasing number of researchers have focused on EVs derived from specific cells. An in-depth understanding of the molecular mechanisms by which different populations of EVs in adipose tissue is important for further understanding how adipose tissue drives cell–cell communication and for discovering biomarkers (Figure 2). We will next detail the role of specific cell-derived EVs in metabolic diseases.

### 3.3. EVs Derived from Adipocyte

Adipocytes are the main cell type in adipose tissue and are primarily used for fat storage [92]. In a new study on mice, researchers described that adipocytes not only release fatty acids but also release EVs-sized, lipid-filled vesicles (AdExos). These AdExos can become a source of lipid for local macrophage, which can regulate macrophage differentiation and function. At the same time, AdExos was also found in blood, raising the possibility of its effects outside of adipose tissue [26]. In recent years, numerous studies have shown that adipocytes are capable of releasing large amounts of functional EVs, but the subtypes and composition of adipocyte EVs have not been clarified. Durcin et al. [93] isolated the subtypes of adipocyte EVs, i.e., small extracellular vesicles (sEVs) and large extracellular vesicles (lEVs), by combining microscopic, biochemical, and high-resolution mass spectrometry (MS) techniques and demonstrated an enrichment of *β*-actin and actin-4 in the lEVs. The enrichment of *β*-actin and actin-4, while major vault protein (MVP) was specifically enriched in sEVs [94]. However, most studies on adipocyte EVs to date have focused on sEVs, which have been found to play an important role in metabolism. Adipocyte sEVs from healthy 3T3-L1 adipocytes increased pancreatic *β*-cell survival and proliferation and promoted insulin release, whereas sEVs from inflammatory adipocytes led to *β*-cell death and dysfunction. Meanwhile, adipocyte-derived sEVs from lean people produced similar beneficial effects, whereas sEVs from obese adipose tissue were harmful to human *β*-cells [28]. Positive or negative functional crosstalk between adipocyte sEVs and pancreatic *β*-cells depends on the pathophysiologic state of the source adipocytes. However, sEVs from obese and insulin-resistant adipocytes can promote compensatory insulin release enhancement in the early stages of T2DM. A recent study using fluorescent tracing and stable isotope labeling with amino acids in cell culture (SILAC) labeling of adipocyte sEVs paired with phosphorylated proteomics found that after adipocyte sEVs transferred functional proteins to *β*-cells, the proteins in the EVs were phosphorylated, which augmented the GPCR/Camp/PKA signaling pathway, and ultimately augmented the glucose-stimulated insulin release of the first phase of mouse pancreatic islets. Whereas such an effect was only present in EVs isolated from obese and insulin-resistant mice [27]. As the disease progresses and adipocyte inflammation increases, the beneficial contribution of adipocyte EVs to glucose metabolism may gradually be outweighed by their deleterious effects, and future studies may consider exploring therapeutic blockade targeting adipocyte EVs or generating adipocyte EV mimics with proinsulinic function for the treatment of T2DM. In a preliminary study in obese patients, multiple regression analysis showed the strongest and most significant correlation between circulating adipocyte EVs and elevated triglycerides, suggesting that adipocyte EVs are associated with lipid metabolism [95]. Consistent with this result, a more recent study found that injection of adipocyte EVs into B6/J-Rab27a-Cas9-KO mice significantly affected fatty acid metabolism in the mice. Lipidomic analysis was also used to show the presence of enzymes related to fatty acid metabolism in adipocyte EVs, including adenosine diphosphate (ADP)-ribosylation factor and mitogen-activated protein kinase-3 [96]. Adipocyte EVs are a promising biomarker of lipid and glucose metabolism with the potential to detect metabolic status in humans, including individuals without metabolic risk factors.

Recent studies have emphasized the importance of adipocyte EVs in metabolic diseases. Adipocyte EVs containing metastasis-associated lung adenocarcinoma transcript 1 (MALAT1) can regulate energy intake *in vivo* and *in vitro* through the hypothalamic mammalian target of rapamycin (mTOR) signaling pathway, and MALAT1 is able to act as a competing RNA to inhibit the function of miRNAs, which in turn affects mTOR signaling, appetite, and body weight [29]. Yu et al. [22] reported that miR-27a in adipocyte-derived EVs may induce insulin resistance in skeletal muscle by inhibiting proline-rich acidic protein *γ* (PPAR*γ*) [19]. Obesity is strongly associated with poor prognosis in patients with advanced colorectal cancer (CRC), and it has been found that increased expression of microsomal triglyceride transfer protein (MTTP) in adipocyte EVs from CRC patients with high body fat ratios acted as an inhibitor of iron pituitary disease and reduced sensitivity to chemotherapy. Mechanistically, the MTTP/ PRAP1 complex inhibits the expression of zinc finger E-box-binding homology box 1 and upregulates glutathione peroxidase 4, leading to a decrease in polyunsaturated fatty acid ratio and lipid reactive oxygen species (ROS) levels [30]. A novel intracellular signaling pathway mediated by adipose-derived EVs was revealed and suggested that treatment targeting released MTTP may reverse CRC resistance to oxaliplatin. Numerous reports have shown that resistin in adipocyte EVs is strongly associated with hepatic steatosis and other fatty liver diseases. Interestingly, the researchers identified that melatonin can reduce the production of resistin included in adipocyte-derived EVs through the brain and muscle Arnt-like protein 1 (Bam 1) transcription inhibition [97]. This study illustrates a novel melatonin-mediated regulatory pathway from adipocytes to hepatocytes. Meanwhile, clinical studies have shown that the levels of miR-7-5p, miR-20a-5p, miR-92a-3p, miR-195-5p, and miR-374b-5p in adipocyte-derived EVs were significantly downregulated after the treatment of T2DM with pioglitazone, in contrast to miR-195-5p, whose changes in miRNA expression were associated with lipolytic inhibition and improvement of insulin sensitivity were closely correlated [98], revealing the potential of adipocyte EVs and related contents as diagnostic targets for metabolic diseases.

### 3.4. EVs Derived from Adipose Immune Cell

During the development of obesity, the expansion of adipose tissue leads to infiltration and activation of immune cells, which in turn triggers a series of inflammatory responses [99–102]. Although adipocytes are the predominant cell type in adipose tissue, innate immune cells such as macrophages, NK cells, neutrophils, eosinophils, and dendritic cells, as well as adaptive immune cells such as T and B cells, synergistically play important roles in adipose tissue to maintain adipose tissue function and homeostasis [103–108]. Macrophages are the most abundant immune cell type accounting for 40%–50% of the total adipose-resident immune cells [109]. Previous studies have found that in adipose tissue, the key event in insulin resistance is the activation and accumulation of proinflammatory macrophages, which influence the metabolic status of adipose tissue parenchymal cells through the release of cytokines, such as IL-6 and TNF-*α*, which induces impaired insulin metabolism pathways and abnormal glucose metabolism, providing a potential cause of obesity-induced insulin resistance [106, 110–115]. However, the limited therapeutic efficacy of using anti-TNF-*α* therapies for improving obesity-induced insulin resistance and glucose metabolism suggests that macrophages in adipose tissue may also influence metabolic status through other pathways. Over the past decade, numerous studies have demonstrated the role of EVs in systemic homeostasis and metabolic disease pathogenesis [116], and more recently it has been found that macrophage-derived EVs can be efficiently internalized by adipocytes, and EVs from LPS-activated macrophages promoted the expression of inflammation-related genes in adipocytes [34]. EVs from adipose tissue macrophages also regulate glucose uptake and mitochondrial activity by targeting NDUFA4 gene expression via miR-210, promoting diabetic obesity in mice [35]. In addition, Ying et al. [36] reported that adipose tissue macrophage-derived EVs in obese mice inhibited insulin signal and glucose tolerance through direct inhibition of its target gene PPAR*γ* by miR-155 [32]. Meanwhile, Zhang et al. [34] found that M1-type THP-1 cell-derived EVs can impair the insulin metabolic pathway in human adipocytes. However, some researchers found that 223 miRNAs were detected to be released into the conditioned medium after treatment of THP-1 macrophages with LPS, suggesting that adipose tissue macrophage-derived EVs can play important functional roles by delivering miRNAs. Accumulation of proinflammatory macrophages in adipose tissue is significantly associated with inflammation in visceral adipose tissue, and one of the main potential causes of obesity-induced insulin resistance is chronic systemic inflammation in visceral adipose tissue, and inhibition of proinflammatory macrophage accumulation in visceral adipose tissue can work well in the treatment of insulin resistance.

Recently, it has been found that NK cells in adipose tissue are significantly associated with obesity-induced adipose stress, macrophage activation, and insulin resistance. Obesity-induced adipose stress leads to the upregulation of ligands for natural cytotoxicity triggering receptor 1(NCR1) on adipocytes, which promotes the activation of NK cells to proliferate, and the release of IFN-*γ* induces macrophage polarization, which leads to glucose metabolism abnormality and insulin resistance, which suggests that NK cells can exacerbate the metabolic disorders through the cytokine release to exacerbate the symptoms of metabolic diseases [117]. EVs often have similar biological functions to their cell of origin, and previous studies have found that NK cells can produce EVs [118, 119], and can alleviate depressive symptoms through miRNAs in EVs [120]. Based on this, we hypothesized that NK cells in adipose tissue are likely to modulate macrophage polarization through EVs, and therefore, the NK cell-derived EVs with macrophage axis in adipose tissue may be a promising new target for patients with metabolic syndrome to reduce the risk of T2DM progression.

### 3.5. EVs Derived from Adipose Stem Cell

Adipose-derived stem cells (ADSCs) are multipotent stem cells present in adipose tissue with properties such as self-renewal, multidirectional differentiation, and immune regulation [121]. ADSCs have been shown to have significant therapeutic effects on metabolic diseases [122–124], but these protective effects are dependent on the paracrine release of ADSCs due to the lower differentiation rate of effector cells compared to ADSCs after transplantation. EVs from ADSCs are key repair factors in metabolic diseases, and ADSCs-EVs are involved in the regulation and repair process of metabolic diseases through the inclusion of bioactive molecules such as miRNAs, proteins, and cytokines [125, 126].

ADSCs-EVs are proved to be effective in controlling obesity-associated inflammation and metabolic disorders. ADSCs-EVs can be internalized by macrophages in adipose tissue. Treatment for obese mice with ADSCs-EVs gets good results by regulating M2 polarization, reducing inflammatory infiltration, and improving insulin sensitivity. In line with this result, it has also been reported that ADSCs-EVs can transport the signal transducer and activator of signal transducers and activators of transduction-3 (STAT3) into macrophages, promote M2-type polarization of macrophages, and improve insulin sensitivity and glucose tolerance in mice on a high-fat diet [38, 127]. Promoting tissue repair and regeneration is also an important approach of ADSCs-EVs to restore metabolic disorders. It has been demonstrated that ADSCs-EVs can ameliorate diabetic nephropathy symptoms by promoting autophagic flux in podocytes and inhibiting podocyte apoptosis through enhanced miR-486 expression [39, 128]. In the past decade, several studies have shown that ADSCs-EVs can promote diabetic wound healing and ischemic tissue angiogenesis in animal models [40, 129–142]. Furthermore, ADSCs-EVs can be designed or engineered to get better therapeutic effects. Glyoxalsse-1 (GLO-1), as a key rate-limiting enzyme in the glyoxalase system, catalyzes the transfer and isomerization of methylglyoxal produced during glycolysis. Overexpression of the GLO-1 gene downregulated ROS in endothelial cells. Zhang et al. [45] enhanced the therapeutic effects of GLO-1 in ADSCs-EVs by overexpressing the GLO-1 gene enhanced endothelial cell migration and angiogenesis in a high glucose environment [41]. The above studies suggest that they can improve blood supply and cell survival in damaged tissues by promoting neovascularization and inhibiting apoptosis, thereby facilitating the tissue repair process. In addition, ADSCs-EVs can modulate metabolic processes by regulating the expression of metabolism-related genes and the activation of signaling pathways, as well as carrying antioxidant molecules such as glutathione peroxidase and superoxide dismutase [143, 144].

ADSCs-EVs possess their own advantages in future applications. Since adipose tissue is one of the tissues widely found in the human body, the origin cells, the ADSCs are easy to obtain by using noninvasive ways. ADSCs-EVs also show good stability and can be stored and maintained biologically active for long periods of time in both *in vitro* and *in vivo* environments [145]. This makes the preparation, preservation, and delivery of ADSCs-EVs easier and more conducive to clinical applications. ADSCs-EVs have low immunogenicity [146]. Compared to their mother cells, ADSCs-EVs can reduce or avoid potential immune rejection. This is important for transplantation or delivery into patients to reduce the risk of immune reactions and related complications [147]. ADSCs-EVs carry a variety of bioactive molecules such as proteins, nucleic acids, and lipids. These molecules can regulate physiological processes, such as cellular metabolism, inflammatory response, and angiogenesis, which can have an impact on the development and treatment of metabolic diseases. The diverse components of ADSCs-EVs allow for a broader range of regulatory roles, which can help to ameliorate the pathological processes of metabolic diseases [148]. It is also important to note that although ADSCs-EVs show potential therapeutic effects in metabolic diseases, they are still in the research stage, and their clinical application still requires further study and validation. In addition, the specific mechanisms and roles of ADSCs-EVs still need to be further investigated to better understand the prospect of their application in metabolic diseases. ADSCs-EVs have many advantages, but each stem cell-derived EVs may have unique advantages in its specific application. Therefore, the specific choice of stem cell-derived EVs may need to be evaluated and selected based on specific therapeutic needs and disease features.

### 3.6. EVs Derived from Adipose Vascular Endothelial Cell

Adipose tissue is highly vascularized, and adipose tissue vascular endothelial cells are a class of cells present in the intima–media layer of adipose tissue blood vessels, which play a key role in the adipose tissue vascular system, regulating adipose tissue function and metabolic processes [149–153]. The high degree of vascularization of adipose tissue implies that there is a close interaction between the vascular and adipose septa, and a recent study has shown that vascular endothelial cells retain pluripotent stem cell-like features and can differentiate into adipocytes, as well as other cell types, under the influence of transforming growth factor *β*2 [154]. The interaction between adipocytes and adipose vascular endothelial cells can be mediated by a variety of cellular signals. VEGF-B was initially described as a vascular growth factor, and recent studies have found that VEGF-B mediates fatty acid transport and insulin sensitivity [155]. VEGF is a direct transcriptional target of HIF-1*α*. Thus, adipose tissue hypoxia may upregulate the expression level of VEGFR2 in endothelial cells, which transduces VEGF-triggered angiogenic signaling. Notably, specific expression of VEGF in adipose tissue ameliorates tissue hypoxia and inhibits fibrosis and local inflammation in adipose tissue [156, 157].

Adipose tissue, especially brown adipose tissue, is the most vascularized tissue in the body, and its adipose vascular system has multiple functions in regulating adipocyte function [158, 159]. Paracrine regulation of adipocyte function is regulated in adipose tissue through the production of various factors and cytokines by vascular endothelial cells, and while adipocyte-derived factors are relatively well characterized in regulating adipose angiogenesis, the mechanism by which endothelial cells regulate the paracrine function of adipocytes is still unclear. In recent years, the potential of vascular endothelial-derived EVs has attracted the attention of researchers, and Caveolin-1 (Cav1), an important membrane-bound structural and signaling protein, is abundant in adipocytes in adipose tissue as well as in endothelial cells. After researchers successfully knocked out the Cav1 gene in adipocytes, Cav1 protein expression was still detected in adipose tissue, and through a newly generated mouse model, researchers found that endothelial cells transferred Cav1-containing EVs into adipocytes, which in turn interacted with endothelial cells by releasing EVs [42]. This study reveals a new form of communication between adipose vascular endothelial EVs and adipocytes, which may function on metabolic regulation, inflammatory response, and regulation of vascular function.

### 3.7. EVs Derived from Other Cellular Sources in Adipose Tissue

Recent studies showed that neuron-derived EVs can contain toxic proteins that contribute to the progression of neurodegenerative diseases [160]. Meanwhile, You et al. [161] suggested that ATPase Na^+^/K^+^ transporting subunit alpha 3 (ATP1A3) isolating from neuron EVs can be a potential diagnostic biomarker of neurodegenerative diseases. At present, there are few studies on neuron EVs from adipose tissue. However, neuronal cells form part of the adipose tissue. The sympathetic nervous system is intimately involved in the development and function of adipose tissue [162–165]. These indicate the research between adipose tissue neuron EVs and metabolic diseases will be a new direction in the future. Preadipocytes and fibroblasts are also present in adipose tissue [95, 166]. Based on this, it is reasonable to hypothesize that preadipocyte-derived EVs play a role in promoting the progression of metabolic diseases. Fewer studies focus on the relationship between preadipocyte-derived EVs and metabolic diseases, and more researches are needed to fully understand it. Emerging evidence suggests that adipose tissue fibrosis plays an important role in the regulation of adipose tissue health. Clinical studies have reported a correlation between the accumulation of extracellular matrix in subcutaneous white adipose tissue and insulin resistance [151]. Importantly, abnormal glucose metabolic status was significantly improved by systemic collagen IV knockdown and adipose tissue-specific inhibition of fibrosis. The same sequencing results indicated the presence of a fibroblast progenitor cell population in vascular adipocytes and fibroblasts to maintain the structure of adipose tissue [167], implying that we can focus on EVs as well as their origin, which may provide new insights for the treatment of metabolic diseases.

## 4. EVs as Metabolic Disease Biomarkers and Therapeutic Potential

EVs have attracted the attention of scientists in the fields of obesity and diabetes for their potential use in diagnostics due to their ubiquitous distribution in various body fluids. In a mouse model, researchers found that obesity was able to alter the miRNA profile in plasma EVs, including increases in miR-122, miR-192, miR-27a-3p, and miR-27b-3p [168]. Meanwhile, monitoring of EVs helps to track obesity, while monitoring TGF-*β*1 carried by plasma EVs helps to monitor T2DM status in obese patients [169]. A recent study using LC-SWATH/MS analysis revealed the presence of potential obesity biomarkers such as UCP1, Glut1, MIF, and copper blue protein in brown adipose tissue EVs [170]. At the same time, researches on white adipose tissue EVs revealed that obese visceral adipose tissue EVs exhibited proteins associated with adipose tissue inflammation and insulin resistance, such as transforming growth factor-*β*1 (TGF-*β*1), Cav1, CD14, mimecan, thrombospondin-1, fatty acid binding protein 4 (FABP-4), and AHNAK nucleoprotein (AHNAK). Thus, adipose tissue EVs have the potential to serve as therapeutic targets and important biomarkers for disorders of metabolism targets and important biomarkers [169].

Clinical studies have demonstrated that systemic insulin resistance in obese and NAFLD patients is associated with adipocyte-derived EVs. It can be used as a therapeutic target for metabolic disorders. In obese patients, high expression of miR-140-5p in adipose macrophage EVs led to elevated levels of prostaglandin-endoperoxide synthase 2 (PTGS2), malondialdehyde (MDA), lipid, ROS, and mitochondrial damage in the organism, which further induced significant cardiac damage [37]. This all implies that our differentiation of EVs of different cellular origins in adipose tissue could serve as an important tool for the diagnosis of metabolic diseases. Despite significant progress in research targeting adipose tissue EVs over the past decade or so, there are still many challenges that need to be addressed in this developing field. For example, as new studies continue to emerge on the presence of multiple types of cargoes in EVs affecting multiple biological pathways, identifying precise, and effective diagnostic targets have become an ongoing problem for researchers.

In addition to the diagnostic potential of adipose tissue EVs, their therapeutic potential should not be overlooked. Increasing evidence indicated the therapeutic effect of EVs processes the potential benefits of easier storage, longer cycle, higher stability, and lower tumorigenicity [171]. An interesting finding describes that microfragmented adipose tissue secretome contains more growth factors and cytokines involved in tissue repair and regeneration, suggesting that EVs from all cells in adipose tissue are more therapeutic compared to when adipocytes are excluded [172]. Meanwhile, many studies have found that treatment of obese mice with adipocyte EVs isolated from lean mice alleviates their symptoms of glucose intolerance and insulin resistance. MiR-342-5p from ADSCs-EVs can protect endothelial cells from atherosclerosis [173]. Moreover, EVs can be engineered to enhance their effects through gene manipulation, biomaterial conjugation, and so on. For example, by reducing miR-140-5p in EVs, researchers attenuated iron prolapse and cardiac damage induced by EVs in obese adipose macrophages. Overexpression of GLO-1 in ADSCs-EVs ultimately promoted angiogenesis by activating the eNOS/AKT/ERK/P-38 signaling pathway and inhibiting AP-1/ROS/NLRP3/ASC/Caspase-1/IL-1*β* [41]. Combining peptide-based hydrogel FHE (multifunctional hydrogel composed of Pluronic F127, oxidative hyaluronic acid and EPL) with ADSCs-EVs efficiency could promote the proliferation and migration of human umbilical vein endothelial cells and facilitate diabetic wound healing [174]. Researches on the therapeutic efficacy of adipose tissue EVs are currently in clinical trials, where researchers have applied adipose tissue EVs mixed with sterile hydrogel to subjects' wounds and found to promote wound healing (NCT number: NCT05475418).

The aforementioned therapeutic strategies rely on cultured cells, such as ADSCs and adipocyte, as a source material for EVs. Notably, according to the latest researches, noncell culture-derived EVs and other biogenic nanoparticles (BiNPs) isolated by using a high robust, pure, and scalable tangential flow filtration (TFF)-based methods have more significant therapeutic benefits. They can obtained from lipoaspirate and are low-cost, high-efficiency, and less time consuming. It has been indicated that BiNPs have promising applications for inflammatory diseases and promoted angiogenesis and exerted a proadipogenic effect *in vivo* [175–177]. At the same time, they can also enhance the expression of VE-cadherin by inhibiting the TRPV4/ROCK1/pMLC2 signaling pathway in the mechanical ventilation model, thereby exerting a protective effect on the pulmonary microvascular endothelial barrier [178]. Despite the tireless efforts of researchers in recent years, there are some challenges to the therapeutic application of adipose tissue EVs. First, there is a lack of a gold standard protocol for high yield, high purity EVs isolation. Second, multiple cells in adipose tissue are capable of releasing EVs, and each cell type has a different function; therefore, further studies comparing EVs from different cell types in the clinical setting are needed to provide optimal choices for the treatment of relevant metabolic diseases. Finally, due to the diversity and complexity of EVs components, further exploration is needed to determine the specific mechanisms of EVs and disease progression and to optimize the targeting of EVs as drug delivery vehicles.

## 5. Conclusions

Adipose tissue is an essential part that maintains homeostasis of energy metabolism in the body and consists of many types of cells. These cells can release EVs to take part in the normal physiological functions of adipose tissue as well as progression of metabolism disorders. The studies on the bioactive cargos, sources, and functions of adipose tissue-derived EVs will provide insights into the role of EVs in metabolic disorders, and, in the future, afford powerful tools for applications of EVs in diagnosis and treatment of metabolic diseases. Enhancing the biological functions of adipose tissue EVs and searching for ways to solve practical problems that EVs face in their application from bench to bedside will finally benefit the patients as well as the EVs industry.

## Figures and Tables

**Table 1:** Role of EVs from adipose tissue and different cells of adipose tissue in metabolic diseases.

Source	Interacting cells	Cargos	Mechanisms and roles	Diseases	References
Adipose tissue	Adipocyte	Mitochondria components	Mitigate the syndrome in high-fat-diet mice	Obesity	[19]
	Adipocyte	NPM3	Regulate the stability of PRDM16 mRNA and enhance the expression of browning-related genes	Obesity	[20]
		miRNAs	Improve glucose tolerance and reduce hepatic FGF21 mRNA and circulating FGF21	Obesity	[21]

Adipocyte	Skeletal muscle cell	miR-27a	Induction of insulin resistance in skeletal muscle by inhibition of PPAR*γ*	T2DM	[22]
	Hepatocytes, skeletal muscle cell	miR-222	Inhibition of insulin receptor-1 expression promotes insulin resistance in liver and skeletal muscle cells of obese mice	T2DM	[23]
	Hepatocyte	Mitochondria-associated proteins	Promotes oxygen consumption in receptor cells, reduces lipid accumulation, and lowers blood glucose	Obesity	[19]
	Adipocyte	Adipokine	Inhibition of insulin-induced AKT phosphorylation impairs insulin signaling in hepatocytes	Insulin resistance	[24]
			Reduced insulin-stimulated glucose uptake	Insulin resistance	[25]
	Cardiomyocyte	miR-802-5p	Targeting HSP60 promotes insulin resistance in cardiomyocytes	Cardiovascular diseases	[26]
		iNOS	*β*3-Adrenergic receptors in brown adipocytes inhibit iNOS-mediated cardioprotection in EVs	Cardiovascular diseases	[27]
	Macrophage		Regulation of macrophage foam cell formation and polarization promotes atherosclerosis	Cardiovascular diseases	[28]
		Lipids	Modulate tissue macrophage differentiation and function	Obesity	[29]
		miR-34a	Inhibition of macrophage M2 polarization promotes obesity-induced adipose inflammation	Obesity	[30]
	Pancreatic *β*-cell	Insulinotropic protein	Enhancement of insulinotropic GPCR/cAMP/PKA signaling pathway to enhance insulin release	Insulin resistance	[31]
		miRNAs	Affects proliferation and function of pancreatic *β*-cells	Obesity	[32]
	Hypothalamic neuronal cell	MALAT1	Inhibits miRNA function, affects mTOR signaling, and regulates energy intake	Obesity	[33]
	Colorectal cells	MTTP	MTTP/proline-rich acidic protein 1 (PRAP1) complex inhibits the expression of zinc finger E-box-binding homology box 1 and upregulates glutathione peroxidase 4 and xCT, leading to a decrease in polyunsaturated fatty acid ratio and lipid ROS levels	Advanced colorectal cancer	[34]

Macrophage	Macrophage		Modulation of adipose tissue function and insulin sensitivity promotes activation of macrophage M1 proinflammatory phenotype	Insulin resistance	[35]
	Adipocyte, muscle cell	miR-155	Inhibition of target gene PPAR*γ* suppresses insulin signaling and glucose tolerance	Insulin resistance	[36]
	Adipocyte, myocyte, hepatocyte	miR-29a	Modulation of obesity-associated insulin resistance	Obesity	[37]
	Adipocyte	miRNAs	Affects adipocyte gene expression, differentiation, and insulin-dependent glucose uptake	Obesity	[38]
		MiR-210	Targeting NDUFA4 gene expression regulates glucose uptake and mitochondrial CIV activation to promote diabetes progression	T2DM	[39]
	Macrophage	miR-222-3p	Modulation of macrophage polarization improves diabetic wound healing	Diabetic ulcer	[40]
	Cardiomyocyte	miR-140-5p	Regulation of glutathione synthesis promotes iron death-induced cardiac injury	Cardiovascular diseases	[41]

Adipose-derived stem cells	Macrophage	Tyrosine hydroxylase	Polarized M2 macrophages and white adipose tissue aggregates attenuate adipose inflammation and obesity	Obesity	[42]
	Podocyte		Enhanced miR-486 expression improves diabetic nephropathy	T2DM	[43]
	Fibroblast	miRNA	Activation of PI3K/Akt signaling pathway promotes diabetic wound healing and inhibits inflammatory response	Diabetic ulcer	[44]
	Vascular endothelial cell	Glyoxalase 1	Activation of eNOS/AKT/ERK/P-38 signaling pathway, inhibition of AP-1/ROS/NLRP3/ASC/Caspase-1/IL-1*β*, and release of VEGF, IGF-1, and FGF to promote angiogenesis	T2DM	[45]

Endothelial cells	Adipocyte	Cav1	Endothelial cells transport Cav1-containing EVs to adipocytes to form Cav1-containing adipocyte EVs	Obesity	[46]

## Abbreviations

EVs: Extracellular vesicles
NAFLD: Non-alcoholic fatty liver disease
T2DM: Diabetes mellitus type 2
HIF: Hypoxia-inducible factor
IL-6: Interleukin-6
TNF-a: Tumor necrosis factor-a
CCL2: C–C motif chemokine ligand 2
iNOS: Inducible nitric oxide synthase
TLR4: Toll-like receptor 4
Nrg4: Neuromodulatory protein 4
VEGF-B: Vascular endothelial growth factor-B
IL-10: Interleukin-10
MVBs: Multivesicular bodies
ILVs: Intraluminal vesicles
ARRDC1: Domain-containing protein 1
ROCK1: Rho-associated protein kinase
PANX1: Pannexin 1
PLEXB2: Plexin B2
NPM3: Nucleophosmin 3
AdExos: EVs-sized and lipid-filled vesicles
sEVs: Small extracellular vesicles
MS: Mass spectrometry
SILAC: Stable isotope labeling with amino acids in cell culture
ADP: Adenosine diphosphate
MALAT1: Metastasis-associated lung adenocarcinoma transcript 1
mTOR: Mammalian target of rapamycin
MVP: Major vault protein
PPAR*γ*: Proline-rich acidic protein *γ*
CRC: Colorectal cancer
MTTP: Microsomal triglyceride transfer protein
ROS: Reactive oxygen species
Bam 1: Brain and muscle Arnt-like protein 1
NCR1: Natural cytotoxicity triggering receptor 1
ADSCs: Adipose-derived stem cells
STAT3: Signal transducers and activators of transduction-3
GLO-1: Glyoxalsse-1
Cav1: Caveolin-1
ATP1A3: ATPase Na^+^/K^+^ transporting subunit alpha 3
TGF-*β*1: Transforming growth factor-*β*1
FABP-4: Fatty acid binding protein 4
AHNAK: AHNAK nucleoprotein
PTGS2: Prostaglandin-endoperoxide synthase 2
MDA: Malondialdehyde
BiNPs: Biogenic nanoparticles
TFF: Tangential flow filtration.
